# Impact of cannabis use and cannabis cessation on inflammation in patients with psychosis

**DOI:** 10.1192/j.eurpsy.2023.583

**Published:** 2023-07-19

**Authors:** B. Romeo, V. Lestra, C. Martelli, A. Amirouche, A. Benyamina, N. Hamdani

**Affiliations:** ^1^Unité de recherche UR Psychiatrie-Comorbidités-Addictions PSYCOMADD, Paris Saclay University; ^2^Psychiatry and addictology, Assistance Publique Hôpitaux Paris, Villejuif; ^3^Cédiapsy, Paris, France

## Abstract

**Introduction:**

The vulnerability-stress-inflammation model is a well-known psychopathological model in patients with psychosis. It implies an imbalance of the microglia activation (M1/M2 pathways’ homeostasis) leading to an over-expression of pro-inflammatory cytokines. However, despite a higher prevalence of cannabis (THC) consumption in patients with psychosis, few studies have investigated the impact of this use on inflammatory markers.

**Objectives:**

The objective of this study was to investigate the impact of cannabis use and its withdrawal on inflammatory markers in patients with psychosis and to explore the link between these inflammatory markers and clinical symptoms.

**Methods:**

A retrospective study was performed including 102 patients with psychosis. White blood cell, hsCRP and fibrinogen levels were measured at baseline and after 4 weeks of cannabis cessation. Urinary THC was also measured at baseline and after 4 weeks of cannabis cessation. Comparisons, adjusted on age, gender, body mass index, smoking status and diagnosis, were performed between cannabis users (THC+) and cannabis nonusers (THC-). To assess the association between inflammatory markers and sociodemographic or PANSS scores, Spearman or Pearson correlations were computed.

**Results:**

After cannabis cessation, a greater increase of leucocyte levels (p < 0.01), monocyte levels (p = 0.05) and a statistical trend to a higher increase of lymphocyte levels (p = 0.06) were found in the consumer group compared to the nonuser group. After 4 weeks of cannabis cessation, higher leucocyte (p = 0.03), lymphocyte (p = 0.04) and monocyte (p < 0.01) counts were found in the THC+ group whereas at baseline no difference was found. A positive correlation was found between monocyte count at 4 weeks and baseline PANSS negative subscore (p = 0.045) and between the variation of monocyte count between baseline and 4 weeks and the PANSS total score at 4 weeks (p = 0.05).

**Conclusions:**

This study shows that cannabis cessation is associated with an increased inflammation depicted by an elevation of white blood cell, lymphocyte, and monocyte levels, which correlates with symptomatology of patients with psychosis. Studying the link between cannabis and inflammation could lead to a better understanding of the pathophysiology of psychosis.

**Disclosure of Interest:**

B. Romeo: None Declared, V. Lestra: None Declared, C. Martelli: None Declared, A. Amirouche: None Declared, A. Benyamina Consultant of: Lundbeck, Mylan, Merck-Serono and Bristol-Myers Squibb , Speakers bureau of: member of board Indivior, N. Hamdani: None Declared

